# Exploring employees’ coping with disability management practices at a South African university

**DOI:** 10.4102/ajod.v12i0.1123

**Published:** 2023-07-25

**Authors:** Aletta M. Moll

**Affiliations:** 1 Department of Psychology of Education, College of Education, University of South Africa, Pretoria, South Africa

**Keywords:** psychological stress, appraisal, coping, coping theory of Lazarus and Folkman, employees with disabilities, disability management, accommodation, workplace

## Abstract

**Background:**

South African legislation promotes the accommodation of employees with disabilities through enabling modifications and adjustments in the workplace. The literature about the experiences of employees with disabilities in higher education environments regarding accommodation is scant. Filling the gap, this research aimed to explore how employees with disabilities at a South African university cope with disability management practices by means of accommodations.

**Objectives:**

The objectives entailed exploring the encounters of employees with disabilities regarding accommodation in the workplace, their beliefs about these encounters and the meaning that the employees with disabilities attached to them.

**Method:**

The study design is grounded in the subjectivist epistemology of social constructionism and took on a qualitative approach. The bounded single-case study concerned formative evaluations. The homogeneous purposive sampling strategy amounted to 13 employees with disabilities. Twelve semi-structured interviews were analysed using thematic analysis.

**Results:**

The participants relied strongly on self-agency to address splintered or unresponsive disability management practices. To avoid marginalisation, they worked extra hard for securing a rightful place at work. Misconceptions of able-bodied peers or managers triggered psychological stress.

**Conclusion:**

Coping with the university’s disability management practices is mainly a stressful challenge, consequently endangering people’s well-being.

**Contribution:**

Exploring the coping of university employees with disabilities through accommodations filled a gap in the literature.

## Introduction

### Background

People are not defined by disabilities – as employees, they are a valuable asset in the workplace (Department Employment and Labour, South Africa n.d.:1). More than a decade ago, Kleynhans and Kotzé (2010:405) conducted a quantitative survey study in a South African province. They found that the attitudes of managers and employees from several companies towards people with physical disabilities in the workplace were negative. Although not stated blatantly, these attitudes came to the fore as apathetic attitudes. Attitude is a tendency to view an entity as more or less favourable, but it can be changed (Blair, Dasgupta & Glaser 2015:665, 681).

This research aimed to explore how employees with disabilities at a South African university cope with disability management practices by means of accommodations, through the lens of coping theory postulated by Lazarus and Folkman (1984). The objectives entailed, firstly, exploring the encounters that the employees with disabilities came across regarding accommodation in the workplace. Secondly, what were their beliefs about the self and the university regarding these encounters? Beliefs relate to how people conceive themselves and their place in the surrounding environment, namely the university. Beliefs shape expectations and, therefore, anticipatory outcomes that are constantly dealt with in appraisals, such as success or failure (Lazarus 1999:71). Thirdly, what meaning or subjective cognitive appraisal did the employees with disabilities attach to these encounters during their evaluation.

### Legislation

Legislation and policies in the Republic of South Africa (RSA) predating this study did not attain changed attitudes towards employees with physical disabilities, according to the study by Kleynhans and Kotzé. The Constitution, Chapter 2, section 9 (RSA, Department of Justice and Constitutional Development 1996) guarantees that people with disabilities may not be unfairly discriminated against, either directly or indirectly. It is enacted by the *Employment Equity Act*, Chapter 2, section 6, subsection 1 (RSA, Department of Labour 1998). Negative attitudes (Kleynhans & Kotzé 2010) as indirect discrimination towards the employees with physical disabilities could have a negative effect on some of these employees. The negative attitudes of managers and employees could be the result of ignorance. The Integrated National Disability Strategy White Paper (RSA, Office of the Deputy President 1997) specifies the raising of awareness and considers it central to the changing of attitudes. The White Paper on the Transformation of the Public Service, Chapter 10, section 10.6 (RSA, Ministry for the Public Service and Administration 1995) prescribes that 2% of government employees must be employees with disabilities by 2005, giving rise to the notion that these employees were not appointed on merit, other than complying with policy.

South Africa also ratified the 2006 United Nations (UN) Convention on the Rights of Persons with Disabilities (CRPD) in 2007. The CRPD promotes inclusive workplaces through the provision of reasonable accommodation, among others, in Article 27. Persons with disabilities is defined in the CRPD as those ‘who have long-term physical, mental, intellectual or sensory impairments which in interaction with various barriers may hinder their full and effective participation in society on an equal basis with others’ (UN 2006:3). Accommodations refer to enabling modifications or adjustments in the workplace, including the ways things are done (Shengli, Paige & Kacey 2022:194). Disability management is the responsibility of the employer’s top management to ensure adequate workspaces by adhering to legislation in the employer’s policy that specifies the relevant practices. Practices entail the full integration of employees with disabilities through accommodations that are effective and practical by taking their individual circumstances into account (Nxumalo 2019:357). Accommodations should, thus, be personalised to increase workplace flexibility and self-sufficiency of employees with disabilities (Padkapayeva et al 2017). As South Africa signed and ratified the CRPD, Article 2 is also relevant, which states that accommodations should be feasible and realistic and not impose ‘a disproportionate or undue burden’ (UN 2006:4). Prior to the CRPD, South African employers were already guided about the inclusion of employees with disabilities and to protect them against unfair discrimination by the Code of Good Practice on the Employment of People with Disabilities (RSA, Department of Labour 2002).

The South African government contributed on various levels to promote the accommodation of employees with disabilities. The National Strategic Framework on Universal Design and Access is one of the initiatives. This framework serves as a prescriptive guide for the promotion and eventual enforcement of universal design and access standards, using a disability inclusion perspective as its motivating force (RSA, Department of Women, Youth and Persons with Disabilities 2021).

However, what are the lived experiences of these employees? The literature about the experiences of employees with disabilities in higher education environments is scant (Waterfield, Beagan & Weinberg 2018:332, 334). The social value of this research is to afford employees with disabilities the opportunity to share their narratives about accommodation in creating awareness of their experiences at a South African university. The scientific value is discussed next.

### Literature review

On the South African front, a qualitative study explored how education, training and development support the wellness of employees with different physical and sensory disabilities at various companies (Van Niekerk, Maguvhe & Magano 2022). The holistic wellness of the employees was considered according to a six-dimensional model of wellness (Van Niekerk et al. 2022:3). The need to ‘develop coping skills’ is discussed as part of the key findings of Van Niekerk and associates (2022:6), thus leading to the question on how employees with disabilities cope in the first instance. As Waterfield and associates (2018:332,334) indicate that there is scant literature about the experiences of employees with disabilities regarding accommodation in higher education environments, the question was further delimited to fill a gap in the literature. Therefore, the empirical research focused on how employees with disabilities coped with disability management practices through accommodations at a South African university.

Experiences that employees with disabilities have to cope with in the workplace are already highlighted in the literature. Employer attitude poses as a barrier regarding accommodation of employees with disabilities (Holness 2016:520; Nxumalo 2019:358; Padkapayeva et al. 2017). An example of a negative attitude is the employer’s fear of the perceived high cost of workplace accommodation (Shengli et al. 2022:195), while the cost of most accommodations is, in fact, inexpensive (Schur et al. 2014:614; Stefan 2002:168).

The employees with disabilities remain marginalised ‘despite various employment laws’ in South Africa that should ensure their full integration in the workplace (Nxumalo 2019:357). Managers’ and colleagues’ lack of knowledge about disability (Padkapayeva et al. 2017; Shengli et al. 2022:194) has adverse effects, and human resource practices are discriminatory against employees with disabilities (Potgieter, Coetzee & Ximba 2017:9). Ignorance, specifically about mental health impairment or conditions, impedes employee accommodations (Holness 2016:510–511, 514; Nxumalo 2019:358; Smith et al. 2022:4–5). Prior to attaining work accommodation, the disability must be declared, which may create negative consequences, in particular for employees with mental health conditions (Shahwan et al. 2022:1253). The Lancet Commission (Thornicroft et al. 2022:1443–1445) states that employees with mental health conditions commonly experience restrictions, such as reduced job opportunities or discrimination at work. Shahwan and associates (2022:1252) mention how stigma about mental health conditions is perpetuated in the workplace. The Lancet Commission calls for an end to all forms of stigma and discrimination (Thornicroft et al. 2022:1438).

Employees with disabilities also struggle with disclosure of their disability to coworkers as well as the frustration that they cannot compete on a level playing field with their able-bodied colleagues (Bam & Ronnie 2020:4–6). They may choose to self-accommodate by figuring out ways of how they can adapt to the work environment, sometimes exerting themselves beyond their physical capacity (Bam & Ronnie 2020:5; Stefan 2002:179; Mkhwanazi & Moll 2022). Shengli and associates (2022:201) refer to ‘employee fear’ of employees with mobility disabilities in disclosing disability and requesting accommodation because of stigma. Employees feared, among others, of being deemed as receiving preferential treatment. Furthermore, some mobility challenges are invisible, despite using a wheelchair. Other concerns preventing employees with mobility disabilities from requesting accommodation included long wait times between requesting and receiving accommodations, bureaucratic red tape, a lack of coordination and carrying out service requests (Shengli et al. 2022:202). In the context of a disability discourse, employees with disabilities in academia are constituted as less capable, less productive and a ‘nonoptimal academic’ (Waterfield et al. 2018:342). Consequently, they have to work hard to be deemed ‘good enough’ when productivity is measured against standards that are the same for all academics (Waterfield et al. 2018:345).

### Theoretical orientation

Filling the gap in the literature – exploring how employees with disabilities coped with disability management practices through accommodations at a South African university – has been framed by the coping theory of Lazarus and Folkman (1984). Coping consists of efforts to ‘manage stressful demands’ (Lazarus & Folkman 1984:134). Coping is defined as process-oriented through constant change in cognitive and behavioural efforts to manage particular demands that are tough or surpassing the resources of the person (Lazarus & Folkman 1984:141). It concerns what people are thinking and doing (Lazarus 1993) when confronted with stressful demands.

Lazarus and Folkman (1984:21) define psychological stress as a relationship between a person (the employee with disability) and the environment (the university as workplace), which is appraised by employees with disabilities as taxing or exceeding their resources and endangering their well-being. Evaluating a person–environment relationship as stressful swivels on subjective cognitive appraisal (Lazarus 1990:4). Psychological stress can set a chain of negative biological physical reactions in the body in motion (Lazarus 1993:48).

Stress is a two-way process between the environment and the individual’s subjective meaning-making of stressors through cognitive appraisals. An encounter between the environment and the person takes place which may or may not cause psychological stress depending on the appraisal. The appraisals are either primary appraisals or secondary appraisals, not to be misconstrued as one is more important than the other – it only indicates the type of appraisals. Coping is not associated with mastery over the environment, as many causes of stress cannot be mastered. No strategy or effort is considered better than the other – any strategy is appropriate if the desired effects are achieved in any given situation, including the effects thereafter (Lazarus & Folkman 1984:134).

Primary stress appraisal of an encounter can be judged as: (1) irrelevant without any implication for a person’s well-being, (2) benign-positive with the promise of preserved or enhanced well-being or (3) stressful. If appraised as stressful, three forms are discerned, namely: (1) harm or loss as damage has already been sustained; (2) threat as anticipated harm or losses and (3) challenge with the potential for gain inherent to the encounter. Examples of work stressors are, respectively, not being promoted (lost career advancement), fear of medical boarding (anticipated harm or loss) and excessive struggles in attaining accommodations (challenge with potential gain).

Secondary appraisal is a judgement or complex evaluative process about what might and can be done by taking all the coping options into account. It questions the viability of whether the outcome will achieve its intended goal as well as what is at stake. Even when people believe they have considerable power to control the outcome of an encounter, if the stakes are high, for example, limited career advancement (Potgieter et al. 2017), any doubt can produce considerable stress (Lazarus & Folkman 1984:35).

## Research methods and design

The study design is grounded in the subjectivist epistemology of social constructionism (Gergen 1985) – an interpretative design – to explore the meaning centred on the subjective process of appraisal (Lazarus 1999:60). In keeping with Lazarus’ (1990) opinion that psychological stress is mainly subjective and objective measures often fall short, this study took on a qualitative approach. The single-case study was bounded, firstly, by the particular group of university employees with disabilities and, secondly, the university policy of disability management regarding practices (Merriam 1998:27). The bounded case study can further be depicted as an evaluative case study that involves ‘description, explanation and judgement’ (Merriam 1998:39). This evaluative case study concerns formative evaluations as the findings are context-specific to this research topic (Patton 2002:220).

As a shared characteristic, the study population consisted of all the employees at a South African university who declared disability. Upon receipt of permission from the University Employee Disability Forum Executive, the University Disability Forum sent an e-mail to all the university employees who had declared disability, inviting them as prospective participants. Using a homogeneous purposive sampling strategy, the sample size amounted to all 13 participants who volunteered, via return e-mail, to be included in the research, thereby obtaining content-rich data via in-depth interviews (Terry et al. 2017). Inclusion criteria were, thus, qualified as participants who had declared their disability at the university under the assumption that they requested accommodations by the employer. An exclusion criterion involved participants with disabilities who declared their disability but did not request accommodation at work. The cohort of participants have long-term physical, mental or sensory impairments sustained prior to or during their employment at the university. Their duration in the employment of the university, at various levels, stretched across decades to a few years.

The participants’ mobility needs ranged from using a wheelchair, crutches, a white cane, transport such as Uber, allocated parking at the university or adapted office furniture. Technological needs in the workplace required software such as digital notetaking for recording plus transcription, speech recognition plus transcription and onscreen visuals converted to speech on headphones, as well as access via the software to the various systems, interfaces and platforms of the university.

The personal needs of the participants included, but were not limited to, additional oxygen supply, medication, adult diapers, carer or personal assistant services, various medical procedures and therapies.

### Data collection

Against the backdrop of a larger multidisciplinary research project, in which the researcher was involved, 13 semi-structured interviews by means of an interview schedule were conducted via the MS Teams platform from June to August 2021, because of the coronavirus disease 2019 (COVID-19) lockdown in South Africa. The recorded interviews were transcribed verbatim afterwards. The project team members, consisting of various academics, could mine data according to their niche areas for individual research purposes.

### Data analysis

**FIGURE 1:**
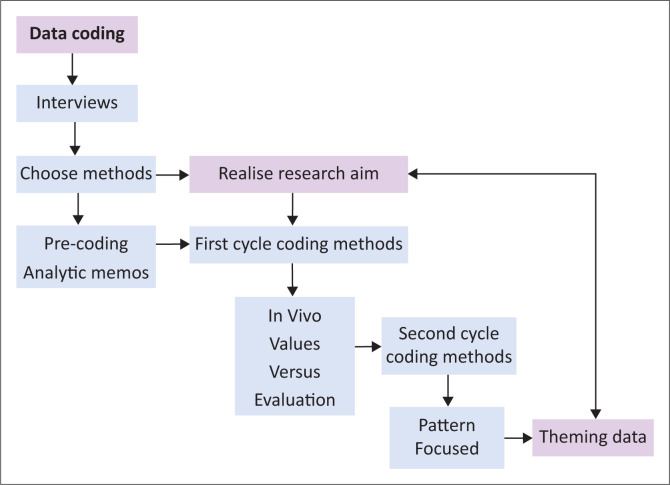
Exposition of the coding process used in thematic analysis.

Numeronyms – containing a number in the abbreviation – as pseudonyms were allocated to the participants starting with P1 (Participant 1) to P13 (Participant 13). Prior to data analysis, these interviews were cleaned to remove all possible identifying particulars. During the cleaning that comprised focused reading, the researcher established that Participant 12 did not request any accommodation other than ordinary support ‘because I am an employee in the university’, similar to the support that every employee would welcome in the workplace. Therefore, only 12 interviews were analysed in accordance with the exclusion criterion. Regarding the second set of cleaned interviews, Figure 1 shows how data analysis proposed by Saldaña (2021) realised the aim of the research. Thematic analysis commenced with manual solo coding of the transcribed datasets using word processing software of Microsoft Word in the table format, which provided columns for the various stages of analysis.

Vis-à-vis trustworthiness, the subjective analysis of data was curbed to a certain extent by: (1) bracketing, including bouncing ideas off another scholar, sometimes on a daily basis, (2) precoding, (3) writing copious analytic memos during the cleaning of each interview and (4) limited unobtrusive member checking via phone. Precoding and analytic memos also assisted in determining the first cycle coding methods that consisted of in vivo, value, versus and evaluation coding of the large datasets of 12 transcribed interviews. Preceding the second coding cycle, recoding took place as also suggested by the iterative framework – a reflexive process – of Srivastava and Hopwood (2009). The second cycle of coding methods consisted of pattern and focused methods, followed by code weaving for theming purposes in realising the aim of the research.

### Ethical considerations

Permission was given, as ethical clearance was obtained from the Research Permission Sub-committee of the Senate Research, Innovation, Postgraduate Degrees and Commercialisation Committee on 22 September 2020. The reference number is 2020_RPSC_010_RS concerning the approval period from 22 September 2020 to 21 September 2022. All procedures involving the participants were compliant with the ethical standards of the South African university involved.

Nonwritten consent, firstly, included disability related to low vision and blindness, as well as limited ability and inability to use upper limbs, and required abilities for signing. Although various software applications may provide assistance in most instances, being respectful and maintaining dignity superseded the requirement of written informed consent. Secondly, upon the informative invitation e-mail, the responding e-mail of the participants presented their implied consent. Thirdly, verbal consent was obtained at the beginning of the interviews. Lastly, the MS Teams platform also displayed in a pop-up message that the interviews were being recorded.

The particular South African university was kept anonymous during the duration of the study.

The topic of the Women in Research project under which funding has been allocated (funding statement) is in accordance with the above, by stating a generic topic, namely ‘Disability management in an Open and Distance Learning workplace’ without identifying the particular university (as per the permission as an ethical clearance document, uploaded as a supportive documentation).

The generic topic (see Data collection) pertaining to the larger multidisciplinary research project in which the researcher was involved according to her individual niche research also included interviews with disability management support staff, health and safety personnel and line managers that do not feature in this submission of individual niche research.

## Results

The participants readily provided situational thick descriptions or content-rich data (Terry et al. 2017) with the primary focus on interpersonal aspects (Denzin 2001:107) during the open-ended interviews. They shared narratives around their encounters regarding accommodation in the workplace; their beliefs about the self and the university practices regarding these encounters, including how these beliefs shaped their expectations and the meaning or subjective cognitive appraisal attached to the encounters and their beliefs during their evaluation of accommodations. Their cognitive appraisals resulted in their coping options. The following themes emerged as shown below.

### Theme 1: Splintered or unresponsive disability management practices resulting in employees with disabilities’ self-agency via various avenues

Metaphorically, the bureaucratic wheels of the vehicle providing accommodations are either turning slowly or have derailed, as evident in the following two quotations that are representative of the participants’ broad evaluation. In coping with ongoing challenges to acquire accommodations, the cumbersome process is taxing and endangering well-being as it is ‘the same story again; there is a lot of hard work, blood, sweat and tears that go into it before eventually getting an outcome’ (P8, M, partially blind). At worst, Participant 11 described disability management as ‘no entrenchment of the policies and procedures in terms of disabilities’ (P11, F, expressive speech impairment) and, therefore, difficult or unlikely to change.

During member checking, Participant 2 compared a senior staff member, concerned with disability management practices, to a wooden door. This inanimate or unresponsive approach was confirmed by Participant 6 who reiterated that ‘personal contact is very important, also for the sake of the dignity of the person with disabilities’ (P6, F, albinism [low vision including nystagmus]). Other than feeling undignified, Participant 6 ascribed unresponsiveness to superiority of staff members dealing with disability management practices versus the inferiority of the employee with a disability:

‘Just knowing that someone *up there* knows that I’m *down here* working with my disability and I’m doing my best and just check on me to see if I need anything and if I’m coping.’ [*Author emphasis*] (P6, F, albinism [low vision including nystagmus])

The emergent interpersonal patterns after data analysis are followed by the participants’ quotations to illustrate the splintered or unresponsive disability management practices at the university.

#### Splintered or unresponsive disability management by way of primary appraisals

As a background to the disability management procedure, Participant 2, a female participant with paraplegia, was confused by the ‘millions of policy documents and procedures in place’, and if an employee with a disability ‘do not know of them [*the procedures or practices*], where to go or which form to submit to HR [*human resources*]’, the employee was lost. Participant 3, a female with a progressive physical disability affecting upper extremities, confirmed that other employees ‘end up not getting assisted because they do not have information. The disability management is an unknown concept … that unclear line of information’ as there are ‘many hoops and loops that you have to jump through, but you have to be persistent’. A ‘dicey issue’ around unresponsiveness is brought to the fore by Participant 3 and supported by Participant 13:

‘Another concern may be the system, where you have to wait a long time and have to write three e-mails or more to get help. So, you need to be persistent and patient, but where do you have the time to be patient when you need immediate help? Some things are urgent.’ (P13, F, paraplegia)

In the case of one participant, efforts to manage a stressful demand amounted to ‘fighting to get this device’. ‘After deciding against a union rep for people living with disabilities … I roped in my lawyer, because I felt that I was being abused by the employer’. (P10, F, progressive motor disease)

Splintered services and revolving offices are visited by employees with disabilities as related by Participant 13. At one stage, wellness services had been outsourced after which the procedure changed without informing employees with disabilities. The pillar to post journey resulted in other employees with disabilities giving up on wellness support as ‘it is difficult now to encourage them to go there’:

‘I went to EE [*Employment Equity*] and they told me that they are also having their own challenges, because they use to report to a certain office, now they are reporting to another office and that is difficult for them … As a staff member with disabilities needing the services, it really doesn’t help … This structural thing and moving departments are messing around with a lot of things here. *[*W*]*e need to know which department is really responsible … because now we don’t know who we should be going to.’ (P13, F, paraplegia)

Splintered or unresponsive disability management practices at the university also affected the participants’ throughput as outlined in the following quotations.

#### Affecting throughput

Unresponsive disability management practices seriously affected Participant 8’s work:

‘At one stage I had to wait for the new upgrade for more than three months and during those three months, because it was an outdated version, I was not able to do my work … Eventually, I did get the new JAWS [*Job Access With Speech*] package, but it’s ridiculous that it took three months.’ (P8, M, partially blind)

Participant 4 reached out for counselling unsuccessfully while her work was negatively impacted:

‘I reached out to [*Employee Assistance*], and I was not successful there. I wrote to [*Employee Assistance*] via e-mail, regarding my own issues with … I did not receive feedback from [*them*] … With the [*mental condition*], I would disappear emotionally, become quiet, not participate in meetings and not willing to take chances. It affects my creativity, as well as in the work environment. As someone who likes change and a creative, it has really affected me and my creativity … There are structures, policies, procedures in place. When it comes to the actual people, I don’t see it working.’ (P4, F, chronic health impairment and consequent post-traumatic stress)

After Participant 5 was denied a single office, she reverted to typing with one hand. ‘Even when I motivated … this recording device needs you to be alone, because I’m making noise for the person I’m sharing with.’ (P5, F, cerebral palsy – hemidystonia [not visually noticeable like hemiplegia])

Participant 6 proposed that ‘it’s not so much about lightening the workload than rather just distributing it differently’:

‘I don’t see why an external marker can’t be appointed to someone who has trouble marking … for when it comes to marking … that’s very tough for me to decipher the students handwriting because I read with a magnifying glass.’ (P6, F, albinism [low vision including nystagmus])

In dealing with the splintered or unresponsive disability management practices at the university, the participants resorted to the following secondary appraisals or complex evaluative processes about what might and can be done.

#### Employees with disabilities’ self-agency by way of secondary appraisals

Participant 6 said: ‘When you join [*this university*] it’s up to you – the person living with a disability – to make things happen.’ (P6, F, albinism [low vision including nystagmus])

#### Relying on immediate able-bodied colleagues’ inherent capacity to do good

In general, the participants considered their close colleagues as their first line of support according to Participants 1, 3 and 5, as shown by the words of other participants:

‘[*Y*]our colleagues … I always have someone in hand to help me with this and that … Collegiality is the key for me … It’s a close-knit department that makes it easier for me.’ (P2, F, paraplegia)

‘The colleagues that I work closely with, know about my condition and are aware of what I can and cannot do or what’s easy for me and what’s challenging for me and they are very accommodating in that sense.’ (P6, F, albinism [low vision including nystagmus])

#### Identifying responsive individuals to assist with disability management practices

Participant 2 described the individual who handles all her disability management matters via the relative department: ‘Having someone like [*name of person*] who brings about warmth into that office, is an invaluable addition’ (P2, F, paraplegia). Participant 1 said: ‘They should get more dedicated people there in their offices. I think they are thinly spread there’ (P1, F, lower extremity amputation). Participant 10 also identified a particular individual and is supported by Participant 9:

‘The only person that I go to and I make sure that I update her … is a lady from the Employment Equity office. She has been a very supportive person. I just want to give her a hug, because it really helped me not to resign …. (P10, F, progressive motor disease)

Participant 10 claimed that as disability management lacks criteria in practice, she also turned to forging relations with other senior colleagues who could influence the practices or implementation of disability management:

‘There are no criteria that the university has in place. What has happened is that, because of the person that I am and the relations that I have built over the years at the institution, the people that are my go-to people now, are the people that I forged relations with …’ (P10, F, progressive motor disease)

#### Helping other employees with disabilities

The participants took it upon themselves to assist other colleagues with disabilities at the university as summarised in the words of Participant 1: ‘I would like to do the same for other people.’ Participant 13 elaborated:

‘Some of the colleagues who are having challenges with disabilities can help each other in terms of getting support from the university … *[*W*]*e are sort of veterans, so we have to help others.’ (P13, F, paraplegia)

Other participants also voiced altruism including the intention to leave ‘a legacy … that I’m supporting people who are differently abled’ (P10, F, progressive motor disease):

‘Tomorrow I’m going to meet someone who’s journey just started and one of the things I told her, is that this is something that you learn how to live with and it’s not a once-off thing.’ (P9, F, incurable health impairment)

‘I have been in contact with two other gentlemen who are disabled employees and they are suffering the same fate that I am suffering … The other day we were speaking about sitting and one of the gentlemen mentioned that his back is sore from sitting on the bed and the wheelchair and … I had two chairs made … and I gave the other one to him. We talk and help each other.’ (P10, F, progressive motor disease)

#### Negotiating accommodations including attitude

In battling with mastery over the environment, Participant 10 provided two options – rent the software or deduct money monthly from salary – in an effort to acquire voice recognition and typing software:

‘The last time that I was in the meeting where I broke down, I was also giving the employer an option to buy the gadget for the institution and rent it out for me or buy the gadget and take the money from my salary every month, if getting me a gadget is such a problem.’ (P10, F, progressive motor disease)

Participant 2 mostly appraised encounters as benign-positive with the potential of preserved or enhanced well-being, as she is of the opinion that ‘it also has to do with one’s attitude as well. It is your whole attitude with yourself and your disability that makes it easier for other people’. ‘Sometimes things are difficult, but I’m more of a positive rather than negative person’ (P1, F, lower extremity amputation). Participant 7’s attitude is that she does not ‘make the problems a limitation as far as she can handle it’ because ‘disability adjustment must come from both sides’. (P7, F, epilepsy)

Participant 13 is of the opposite opinion:

‘Rather push and if they don’t respond, then you just have to be crazy for things to happen. Sometimes it takes a person being crazy for them to listen.’ (P13, F, paraplegia)

### Theme 2: Avoidance of marginalisation resulting in employees with disabilities working extra hard to earn a rightful place in the work environment

The general belief of how the participants conceived themselves in relation to the work environment is aptly embodied in following quote: ‘You start to feel like you don’t have a place at the workplace’ (P8, M, partially blind). Another participant shared a similar sentiment referring to top management: ‘People with disabilities are sort of an afterthought’ (P1, F, lower extremity amputation), reverberating in the employees with disabilities’ belief of getting ‘a favour by employing them [*employees with disabilities*] because we [*the university*] need at least two percent of our staff to be people with disabilities’ (P6, F, albinism [low vision including nystagmus]). Participant 13 asserted that her appointment was on merit despite her disability while colleagues only later ‘realised that I have that skill’ when they became aware of ‘the contribution that I was making’. However, ‘when you apply for a job … in as much as you have a disability, you still want to get a merit appointment*’.* (P13, F, paraplegia)

Participant 10 who became disabled while in the employ of the university took a strong stand against medical boarding:

‘So, if you tell me that … at the end of the day, I can simply go on ill-health and still earn a salary, that is not assisting me; that is killing me instead. Right now, my work is one of the things that keeps me going, it’s one of the things that still make me see value in myself … pushing me out with ill-health is not solving the problem … I’m meeting my targets … I’m continuing to drive my boat and it’s going in the right direction.’ (P10, F, progressive motor disease)

Marginalisation also became apparent through applying the same performance benchmark as for able-bodied colleagues.

#### Gauged by the same benchmark as able-bodied employees

Participant 5 lamented that ‘they expect me to perform as able-bodied people’ as ‘I’m using one hand to type, but I was expected to work as a person who uses both hands’. Nonetheless, ‘I was always the first to submit work’. (P5, F, cerebral palsy - hemidystonia [not visually noticeable like hemiplegia])

The participants compensated by working extra hard to earn a rightful place in the work environment: ‘It makes you want to prove yourself. So, everything that you are doing, you have to work five times harder’ (P13, F, paraplegia). In maintaining throughput, when one particpant feels well, ‘I push a lot of work and then hold on to it, so that in the days when I’m not feeling well, I … produce what I have already done’ (P13, F, paraplegia).

When Participant 10 was unexpectedly hospitalised:

‘I phoned and briefed my [*superior*] and to my surprise, he went on asking about reports … telling me about situations and telling me to manage the situations’ whereafter she complied by devising a ‘support structure’. (P10, F, progressive motor disease)

Participant 6 adjusted her approach to match meeting practices incompatible with her disability while also embodying Participant 7’s attitude to personally deal with limitations:

‘I rely a lot on my memory because I can’t see well [*albinism including low vision*]. I always make sure that I prepare very well for a meeting and try to memorise the agenda, because they normally use projectors to put these things on and because of my light sensitivity, I can’t see there. I also rely on my hearing a lot, so I just follow the conversation with my hearing.’ (P7, F, epilepsy)

### Theme 3: Misconceptions of able-bodied peers or managers regarding disability and accommodations causing stress to employees with disabilities

Although accommodations ‘give people with disabilities some self-worth, the feeling that they can still do something, still contribute, make a difference and that they are valuable’ (P1, F, lower extremity amputation), able-bodied colleagues or managers sometimes clouded these feelings by being judgemental or setting conditions for work performance. For example, Participant 8 came to office with his white cane. Because of mobility training at the School of the Blind that he attended as a child, on condition that he is familiar with his surroundings, ‘I can walk around without my white cane’ (P8, M, partially blind). Consequently, his colleagues assumed that he could see.

#### Doing things differently versus absconding work obligations

Participant 6 viewed herself as:

‘[*doing*] things differently … with devices and technology [*and not using disability*] as an excuse to not do something [*provided that*]they see that you bring your part. I think working from home … is not for the sake of convenience … I can rather use that time to work [*than struggle with transport because of low vision*].’ (P6, F, albinism [*low vision including nystagmus*])

To the contrary, Participant 8, partially blind with 5% vision in both eyes, was taken aback:

‘At one stage, because I don’t use a screen, I only use earphones with my speech software, some of the colleagues went to the manager and said I’m not working because my screen is off, whereas the stats showed otherwise.’ (P8, M, partially blind)

Participant 5 was reminded not to neglect her duties despite being on sick leave: ‘There were calls that stated that they know that I’m on leave, but I must remember that I am employed by [*this university*], so, when they call, I must answer’ (P5, F, cerebral palsy - hemidystonia [*not visually noticeable like hemiplegia*]). Participant 4 who is accommodated by additional sick leave depicted judgement as absconding work obligations from line managers and is supported by P9:

‘I somehow have experienced that my line management does not really consider this [*mental condition*] or see this as real … Some might see this as a cop out from doing work … They think you are faking it, get over it, go outside, look at how beautiful it is outside … not understanding that it is not about the sun. “Get over it, don’t be so down” … They think: No, I just don’t want to work or I am being lazy.’ (P4, F, chronic health impairment and consequent post-traumatic stress)

‘Especially line managers don’t really understand the condition and they really don’t know how to manage a person that has got a mental health problem. Sometimes the way they try to manage it, is actually making it worse.’ (P9, F, incurable health impairment)

#### Favouritism and stigmatisation

Although Participant 13 has debilitating visible mobility challenges, able-bodied colleagues frowned upon an accommodation to knock off work early:

‘It creates a stigma from other employees … The line manager said we knock off at 4 p.m., but I may leave at 3:30 p.m. so that I have enough time. With that special treatment, people didn’t understand for some time when I would leave early, but they will catch up with me on the way to the taxi spot. So, it’s not like I’m being favoured.’ (P13, F, paraplegia)

Participant 9 living with a serious invisible health impairment found it insensitive when questioned about her reserved parking close to the building: ‘They will say that I am not disabled because I am not in a wheelchair’. Participant 6 considered it ‘an us and them thing’.

## Discussion

A quotation of a participant that connects most of the encounters, beliefs and ultimate meaning-making by the employees with disabilities is stated as: ‘There are structures, policies, procedures in place. When it comes to the actual people, I don’t see it working’. It shows that many university staff members who must execute the disability management practices essentially posed a challenge to the employees with disabilities’ well-being. Ignorance about disabilities contributed to apathetic or negative attitudes and resulted in unresponsiveness regarding accommodation, affecting the participants’ throughput negatively. Lack of clarity about access to the splintered services was also a barrier. In response, the participants devised various ways in attaining accommodations and they worked extra hard to avoid workplace exclusion. Misconceptions of able-bodied peers or managers regarding disability and accommodations caused psychological stress to the participants.

Employees with disabilities coping with the university’s disability management practices is mainly a stressful challenge by means of primary appraisal, in particular when anticipating excessive struggles in attaining accommodations, which refers to challenges with potential gain (Lazarus & Folkman 1984). Staff members of the various divisions at the university concerned with accommodation are in the employment of the university. Thus, top management has the ultimate responsibility in executing disability management practices. As legislated by South African law and embodied in governmental policies, the learned minds at the university’s top management may not sustain discrimination (Potgieter et al. 2017; RSA, Department of Justice and Constitutional Development 1996; RSA, Department of Labour 2002). They also may not sustain hardships (Shengli et al. 2022) and workplace exclusion of employees with disabilities (United Nations 2006). The attitudes of managers and staff members concerned with disability management were still more often than not apathetic (Kleynhans & Kotzé 2010) and remained a barrier regarding accommodation (Holness 2016; Nxumalo 2019; Padkapayeva et al. 2017). The perceived high cost of workplace accommodation as example of a negative attitude (Schur et al. 2014; Shengli et al. 2022; Stefan 2002) is evident in a participant’s appeal to either rent her the software or deduct money monthly from her salary.

In contrast to the study by Kleynhans and Kotzé, negative attitudes of immediate colleagues reduced considerably in this study as many of them became trusted support, although others’ lack of knowledge about disability (Padkapayeva et al. 2017; Shengli et al. 2022) still had adverse effects. A participant confirmed that ignorance, specifically about mental health impairment or conditions, impedes employee accommodations (Holness 2016; Nxumalo 2019; Smith et al. 2022; Thornicroft et al. 2022). The participant related that her throughput was affected and that she was being judged as lazy. Lack of intervention can increase the risk of negative work outcomes (Van Hees et al. 2022:173–174). Although stigma about mental health conditions as invisible disability is perpetuated in the workplace (Shahwan et al. 2022), visible disabilities do not escape stigma as stated by participants and the literature (Shengli et al. 2022; Waterfield et al. 2018).

Acquiring assistive devices remained a taxing challenge despite the Code of Good Practice on the Employment of People with Disabilities (RSA, Department of Labour 2002), resonating to a 2011 finding that ‘technological aspects of the Code were largely unacknowledged, with little effort being made to accommodate the needs of persons with disabilities’ (Jakovljevic & Buckley 2011:55).

According to the participants’ beliefs, they remained marginalised or excluded ‘despite various employment laws’ in South Africa that should ensure their full integration in the workplace (Nxumalo 2019). They are more likely to be graded as ‘working poor’ (Darcy, Taylor & Green 2016) or less likely to be paid employees (Lewis, Dobbs & Biddle 2013). One of the reasons for lower employment than the stipulated at least 2% of staff members that should comprise employees with disabilities (RSA, Ministry for the Public Service and Administration 1995) is that ‘people with disability are discriminated against’ (Darcy et al. 2016). Some of the participants became frustrated or stood aghast that they were expected to perform as able-bodied employees (Bam & Ronnie 2020; Waterfield et al. 2018). Thus, the participants worked extra hard to earn a rightful place in the work environment to prevent being viewed as less capable, less productive and a ‘nonoptimal academic’ (Waterfield et al. 2018). The participants were stigmatised because of their supposed inability to compete with able-bodied colleagues (Darcy et al. 2016; Shahwan et al. 2022; Shengli et al. 2022; Thornicroft et al. 2022; Waterfield et al. 2018). In fending for other employees with disabilities, social cohesion became apparent.

### Recommendations

As disability management practices left much to be desired, according to the participants’ appraisals, readdressing these practices should cover the following:

- Compile a single comprehensive online publication containing all the relevant links for both line managers and employees with disabilities regarding the process of obtaining accommodations. All units and services available should be outlined.
- The online publication should include all the relevant contact details of responsible individuals, frequently updated by the webmaster, and all the relevant forms to be completed, plus specifications, if any, for obtaining various accommodations as well as a function for uploading required motivational documents.
- Any submission by line managers or employees with disabilities or both should be followed by an automated reply acknowledging receipt and containing the procedures involved in the application, as well as relevant contact details including a tracking and tracing number to monitor the progress of the application.
- Applicant employees with disabilities should be informed via return e-mail of outstanding requirements.
- Should the application be delayed, the software programme should automatically flag the concerned superior about the delay, who should then prioritise the matter to the relevant department or departments and monitor feedback.
- Obtain an automated immediate brief survey of employees with disabilities’ feedback following a successful or otherwise application, to determine efficiency or lack thereof.
- Incorporate above employees with disabilities’ feedback as part of internal quality assurance in the university assessment of disability management practices.
- Present certified compulsory continuous professional development sensitivity training for top management, staff members dealing with disability management practices and line managers, coupled with employees with disabilities’ evaluative feedback.
- Conduct further research pertaining to the experiences of employees with disabilities in higher education environments regarding accommodation. This must be in line with the National Strategic Framework on Universal Design and Access.

## Conclusion

There are many challenges that a person with disabilities must go through to secure employment and to keep it. Disabilities can, but do not have to, limit an employee’s capability to perform well. Employment specialists have the task to ensure that employees with various disabilities are fully accommodated to enable their workforce participation. Providing a less stressful workplace for employees would make a difference in changing the dynamic of the organisation.

Employees with disabilities’ coping with the university’s disability management practices is mainly a stressful challenge by means of primary appraisal, consequently endangering well-being. It refutes South African laws, embodied in policies, and the ratified international CRPD.

By exploring the coping of university employees with disabilities through accommodations, a gap in the literature was filled. The value of the research lies in the provision of directions that the university must now follow when appointing employees with disabilities. The methodological limitation of this study is that in-depth interviews were used as the primary and only source of data for developing this case study.
